# A predictive machine learning approach for microstructure optimization and materials design

**DOI:** 10.1038/srep11551

**Published:** 2015-06-23

**Authors:** Ruoqian Liu, Abhishek Kumar, Zhengzhang Chen, Ankit Agrawal, Veera Sundararaghavan, Alok Choudhary

**Affiliations:** 1 EECS Department, Northwestern University, Evanston IL, USA; 2 Oak Ridge National Lab, Oak Ridge TN, USA; 3 Aerospace Engineering, University of Michigan, Ann Arbor MI, USA

## Abstract

This paper addresses an important materials engineering question: *How can one identify the complete space (or as much of it as possible) of microstructures that are theoretically predicted to yield the desired combination of properties demanded by a selected application*? We present a problem involving design of magnetoelastic Fe-Ga alloy microstructure for enhanced elastic, plastic and magnetostrictive properties. While theoretical models for computing properties given the microstructure are known for this alloy, inversion of these relationships to obtain microstructures that lead to desired properties is challenging, primarily due to the high dimensionality of microstructure space, multi-objective design requirement and non-uniqueness of solutions. These challenges render traditional search-based optimization methods incompetent in terms of both searching efficiency and result optimality. In this paper, a route to address these challenges using a machine learning methodology is proposed. A systematic framework consisting of random data generation, feature selection and classification algorithms is developed. Experiments with five design problems that involve identification of microstructures that satisfy both linear and nonlinear property constraints show that our framework outperforms traditional optimization methods with the average running time reduced by as much as 80% and with optimality that would not be achieved otherwise.

Material selection has traditionally been carried out with property cross-plots, a graphical representation of material–property–performance relationships popularized by Ashby^1^. For example, density versus strength plot of different materials can be used by an aircraft engineer to find that for the same strength requirement, a titanium alloy weighs much less than a steel alloy. The disadvantage of chart- or plot-based selection is that only materials with known property are included. It is common knowledge that even within the selected alloy system, microstructural variability leads to a large range of material properties. We are currently moving towards a new paradigm of microstructure–sensitive design^2^ where it is crucial to identify the very microstructure within an alloy system, out of innumerable candidates, that leads to a desired property or a combination of desired properties. Mathematical search-based designs explore the hypothetically infinite space and offers the freedom of engineering unknown microstructures. But the efficiency of mathematical search deteriorates quickly as the candidate space grows, with a microstructure represented by hundreds even thousands of dimensions traditional searches for microstructure design can be slow. In this paper, we explore advanced data oriented techniques to enhance the mathematical search by statistical heuristics, for the purpose of fast and accurate microstructure optimization for a recently discovered alloy, Galfenol.

Galfenol has been shown to exhibit magnetostrictive strains up to 400 ppm in single crystal form (more than 10 times that of *α*-Fe). When a magnetic field is applied to Galfenol single crystal, the boundaries between the magnetic domains shift and rotate, both of which cause a change in the material’s dimensions. This behavior, termed magnetostriction, has been used to transduce magnetic field to mechanical force in micro–scale sensors and actuators^3^,^4^,^5^,^6^. While single crystals of Galfenol provide large magnetostriction, their preparation is expensive. Thus, development of polycrystalline Galfenol with favorable properties for various applications^7^,^8^,^9^ is desirable. A combination of high stiffness, magnetostrictive strains and yield strength is optimal for use in a cantilever beam device that can be used to generate sonar waves (as actuators), measure vibrations (as sensors) or generate electricity (as energy harvesting devices).

While theoretical models for computing properties given the microstructure are known for this alloy, inversion of these relationships to obtain microstructures that lead to desired properties is challenging. The microstructure design of polycrystalline Galfenol can be performed by tailoring the distribution of various crystal orientations (‘the orientation distribution function (ODF)’) in the microstructure (Fig. 1a 10). The structural optimization is carried out along different crystallographic directions to attain favorable properties. The multiple crystallographic directions embedded in the multi-dimensional ODF are used as control variables and the theoretical functions for properties are the objective. The main challenge is to address the following three issues,

(a) High dimensionality: to search in a hypothetically unlimited space of all possible crystal orientation distributions, and converge within a reasonable time.

(b) Multi-objective: to optimize under a requirement of multiple extremal properties that are often in conflict.

(c) Solution completeness: to identify the complete space (or as much of it as possible) of microstructures when more than one solution exists that produce the same optimal property.

Very few published works in literature discuss such design problems. Significant contributions in this area include^11^ where the authors design an ODF that maximizes the deflection of a beam without plastically deforming it. In^12^, the authors design a plate with a circular hole subjected to an in-plane tensile load so as to maximize the load carrying capacity while avoiding plastic deformation. These analyses employ a reduced spectral series representation of the texture that lies in a significantly smaller search space. However, much information in the texture is lost in the reduced representation, and the optimization search is done rather manually, by numerically interpolating the microstructure space to find the location of best performance. Traditional optimization techniques used to search of the answer lead to an unique microstructural solution, rather than the complete space of optimal microstructures. Multiple solutions are favored in the sense that traditional low–cost manufacturing processes such as forming and heat treatment can only generate a limited set of microstructures^7^,^8^,^9^, and a single design solution may not be economically feasible to manufacture^10^. Other optimization techniques that may lead to multiple solutions (such as combinatorial search methods and evolutionary methods) have been explored in materials selection and structural optimization design^13^,^14^,^15^,^16^. However, these methods are often prohibited by the high dimensionality of search space (curse of dimensionality^17^).

Herein, we propose the employment of modern machine learning (ML) techniques as a tool to explore multiple design solutions and diminished searching time in high dimensional microstructure design problems, where the number of distinct design candidates is indeed infinite. Two crucial ML steps, namely, search path refinement and search space reduction, are designed to develop heuristics that tour the search force to a much smaller preferable space. As the diagram in Fig. 2 suggests, the ML method (bottom route) has these two steps (marked as 2 and 3) executed laterally, after a data preparation step (marked as 1) that precedes. The three steps supplement a traditional direct-search method (top route) by performing a search space preprocessing, before the actual search goes into action. Such a ML-based preprocessing is designed to locate critical regions of a search space with a small overhead, so that the search force can be consciously concentrated.

## Results

### Design Problems

We start with a spectral representation of the microstructure and its relevant statistics and present five polycrystalline alloy design problems, each with a different property objective to optimize. The properties are either a singular or a composite of the following: Young modulus (*E*), yield strength (*Y*) and magnetostrictive strain (*m*_*s*_). For sensor applications where Galfenol is used in the form of compliant beams, *E* is inversely proportional to deflection, so a lower modulus results in higher deflection for the same applied stress. Higher yield strength *Y* will increase the load bearing capacity of the structure and it is our objective to maximize *Y*. A higher value of magnetostrictive strain (*m*_*s*_) will enhance the capability of material to act as a sensor material.

Two additional composite functions are designed to express the need of a set of properties balanced for peak performance, fulfilling the requirement of multi-objective design. Composite function *F*_1_ = *Y* ⋅ *m*_*s*_/*E* is maximized to search for a microstructure with a low modulus and high value for strength and magnetostrictive strain. Another function *F*_2_ is given by a combination of two desired properties, stiffness component 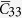 = 274.94 *GPa* and magnetostrictive strain *m*_*s*_ = 99.53 *ppm*. A weighted Gaussian function of the form *F*_2_ = 10exp(−3(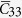 − 274.94)^2^ − 2(*m*_*s*_ − 99.53)^2^) is optimized such that the maximum value of *F*_2_ = 10 is attained at the desired property values.

### Property Calculation

**Figure 1:**
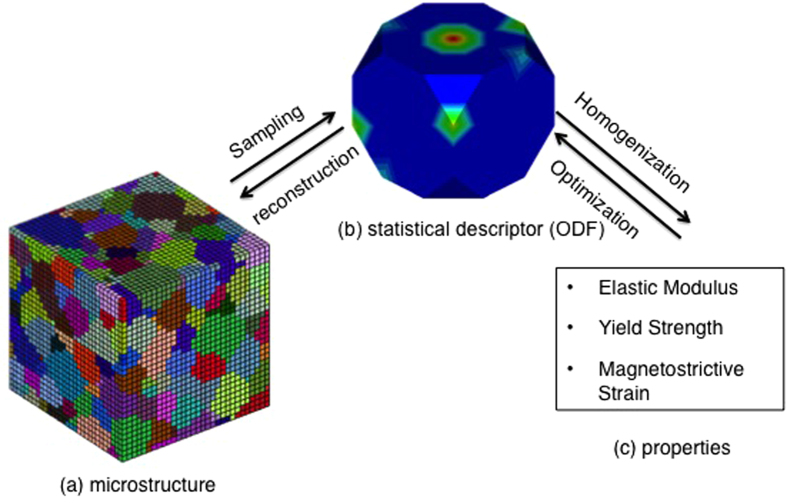
Microstructure representation of Galfenol. (**a**) Polycrystalline microstructure of Galfenol, with colors denoting different crystal orientations. (**b**) ODF (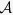) for given microstructure. (**c**) Various properties estimated using homogenization technique from the ODF.

The orientation distribution function (ODF), the probability density function for orientations, is applied for the quantification of crystallographic texture of Galfenol, as seen in Fig. 1. An axis-angle parametrization of the orientation space proposed by Rodrigues has been used. This is based on the unique association of an orientation with a rotation axis ***n*** and an angle of rotation *θ* about the axis such that ***r*** = 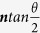. Relationship of ***r*** with the standard rotation matrix that maps the sample axes to the crystal axes is given in Section 2 of^10^. The ODF (in Fig. 1b) denoted by 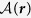 represents the volume density of crystals with orientation ***r***. If the orientation-dependent property for a single crystal *χ*(***r***) is known, any polycrystal property can be expressed as an expectation value or average given by:


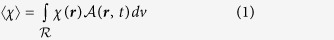


#### Polycrystal moduli calculation

Values of elastic parameters for BCC Galfenol crystal are *C*_11_ = 213.0 *GPa*, *C*_12_ = 174 *GPa*, *C*_44_ = 120 *GPa*. The polycrystal stiffness, 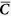, is computed through a weighted average (over 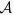) of the stiffness of individual crystals expressed in the sample reference frame. The elastic modulus (along x-axis) is computed through this polycrystal stiffness as^18^:


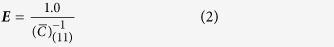


#### Magnetostrictive strain calculation

Magnetostrictive strain in Galfenol single crystals is specified using two independent parameters, λ_100_ and λ_111_, which characterize the changes in normal strain along the 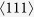 and 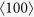 direction resulting from the rotation of a magnetization state into these directions. The magnetostrictive strain tensor for a crystal with magnetization direction given by the unit vector *m* = (*m*_*x*_, *m*_*y*_, *m*_*z*_) (in the crystal coordinate system) is then stated by the following expression:


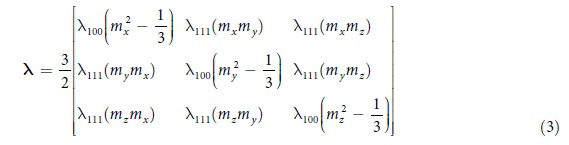


We use the model from Armstrong^19^ which represents the free energy as a sum of internal and external energy terms. The following form of internal energy is taken:


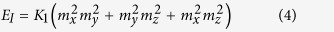


The simple form for *E*_*I*_ used here ensures that a domain in the crystal has minimal and maximal energies when oriented, respectively, along the 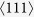 directions (easy direction) and the 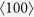 family (hard directions). Application of an external magnetic field leads to an energy change in energy proportional to the intensity of the magnetic field, ***H***, the magnetization of the domain, ***M***, and the direction between them. The direction of the applied magnetic field is represented as ***n*** = (*n*_*x*_, *n*_*y*_, *n*_*z*_) in the crystal coordinate system.


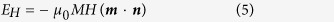


The energy contribution (per unit volume) associated with the interaction of externally applied stress tensor (***σ*** in crystal coordinate frame) with magnetostrictive strains is given as:


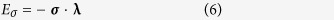


In an ideal crystal without defects (at T = 0K), the domain would align in the direction of minimal energy. However, domain magnetization is expected to follow a Boltzmann–like distribution at higher temperatures due to an increase in entropy. The probability, *P*, that the magnetization direction is equal to ***m*** is given as:


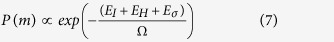


The parameter Ω represents the spread of the magnetization direction from the ideal direction (of minimal energy). The magnetostriction strain tensor *ε* is obtained by averaging the strains over the probability density of magnetization in the crystal.


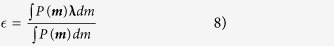


The above integral is calculated by sampling over all possible magnetization directions (ie. all points on a unit sphere). The strain tensor *ε* in the crystal coordinate system is rotated back to the sample coordinate frame *ε*_*s*_(***r***) = ***R***^*T*^***εR***. The overall strain tensor is obtained by averaging *ε*_*s*_(***r***) over the ODF using equation (1). The magnetostrictive strain 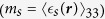 that is optimized in our machine learning approach is the polycrystal averaged strain along the z–direction as measured with respect to an initial unstressed crystal.

For single crystal Galfenol, the various parameters used are as follows: *K*_1_ = 3.6*e*4*Jm*^−3^, λ_100_ = 170 *ppm*, λ_111_ = −4.67 *ppm*, *M* = 1.83/*μ*_0_*Am*^−1^ (*μ*_0_ = 4π × 10^−7^) and Ω = 625*Jm*^−3^ calibrated in^6^. In the examples, we apply the magnetic field of −500 Oe and a compressive pre-stress of 80 MPa, both along the [001] sample direction. At these values, the single crystals achieve a saturation magnetostrictive strain that compares well with the model in^6^. In the examples, the magnetostrictive strain (*m*_*s*_) along the [001] sample direction is optimized. Note that magnetostrictive strain has significant anisotropy, with the saturation strains along [012] direction being 25% lower than that of the [001].

#### Yield strength calculation

The crystal plasticity model described in Method section and also in^10^ is used to calculate the yield strength at all nodal points in the fundamental region. The model adequately captures the macroscopic tensile mode stress-strain response at room temperature reported in^20^ as shown in Fig. 3(b). To further validate the microstructural model, we compared the crystallographic textures seen in BCC iron rolling processes and textures predicted by our model. Fig. 3(a) shows that the model captures both *α* and *γ* texture that arise from rolling of BCC metals (experimental result from^21^). The strength (*Y*(***r***)) at orientation ***r*** is found as the offset z–stress resulting from an applied z–strain of 0.2% under the following velocity gradient^18^:


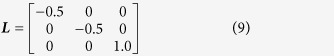


The overall yield strength is obtained by averaging *Y*(***r***) over the ODF using equation (1).

The single crystal properties for elastic modulus, magnetostrictive strains and the yield strength obtained from the above analyses can be visualized on the ODF mesh in Rodrigues space. The plots shown in Fig. 4(left) depict the surface contours with internal slices of the ODF shown alongside in Fig. 4(right). The single crystals with maximum and minimum properties and their locations can be seen directly from these plots. For example, the single crystals with maximum magnetostrictive strains are all located along the z–axis of the Rodrigues space as seen in Fig. 4(a) (right). This corresponds to the z–axis 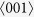 fiber in which the crystal direction of easy magnetization ([001]) is aligned along the measurement axis (sample z–axis).

### Optimization Result Analysis

**Figure 2:**
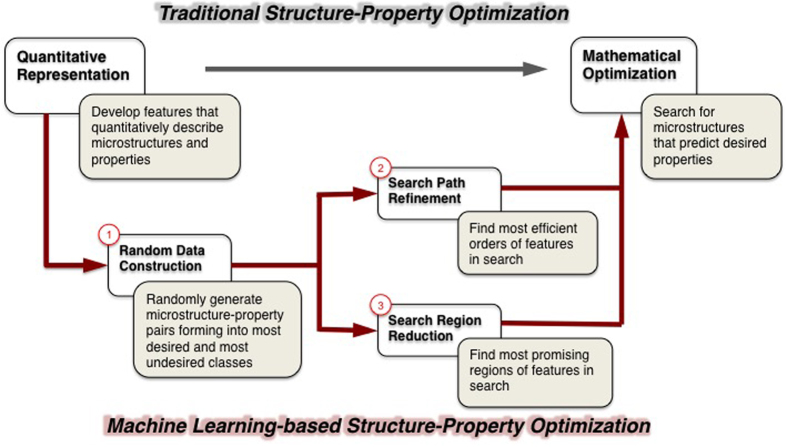
Framework of material structure optimization. The flow on top is the traditional search-based mathematical optimization method. The bottom is the machine learning based method we propose. Three additional steps are inserted to learn a refined and reduced search space.

**Figure 3:**
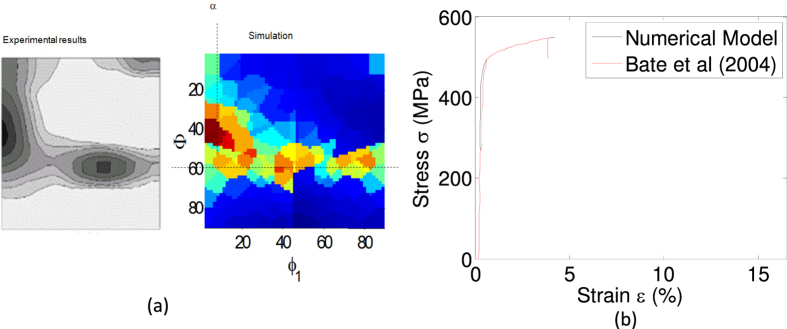
Microstructural model validation. (**a**) Comparison of textures (Euler angle space, *ϕ*_*2*_ = 45°) predicted by our model with experiments on BCC iron reported in^21^. (**b**) Comparison of results of current model with published results in^20^. The plot shows tensile test curves of as-cast polycrystalline Galfenol (alloy composition *Fe*_82.17_*Ga*_16.83_ with 0.5–1% Boron) at room temperature.

**Figure 4:**
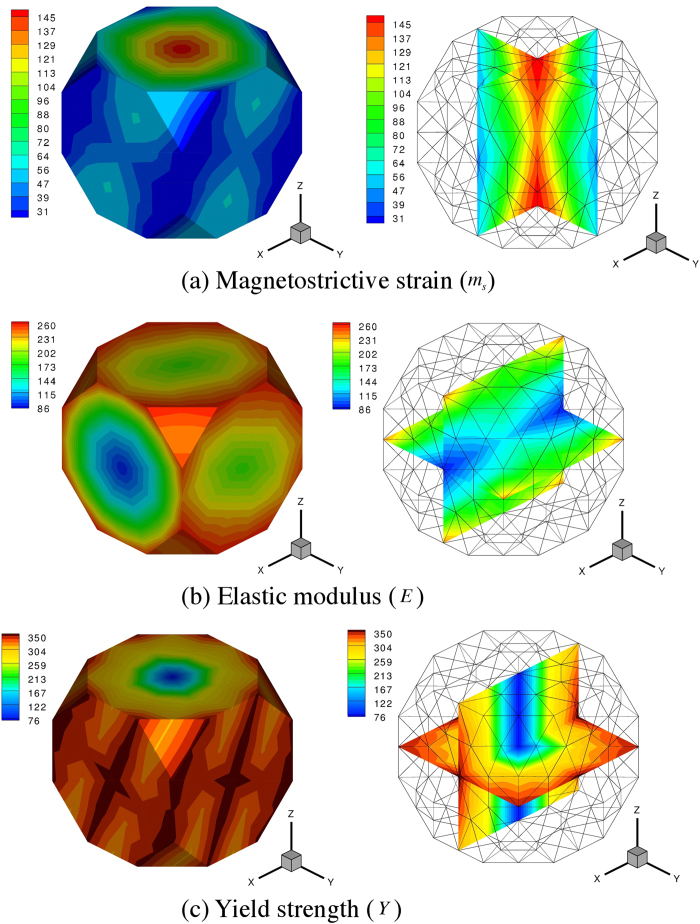
The single crystal properties for elastic modulus, magnetostrictive strains and the yield sgth obtained from our analyses are visualized on the ODF mesh in Rodrigues space. Both the surface contours and internal slices of the ODF are shown. The single crystals with maximum and minimum properties and their locations can be seen directly from these plots.

With the five design problems, the ML-based optimization approach, essential procedures shown in Fig. 2 (bottom route) and each step further explained in the Method section, develops heuristics that guide the search into most promising areas. To demonstrate its superiority at optimality, time efficiency and solution completeness, we compare with three baseline methods, all of them evaluated using three criteria: 1) the optimality, or goodness or property, determined by the sheer value of the property obtained; 2) the efficiency, judged by the time taken for obtaining the result under the same computational environment, the lower the better; and 3) the completeness of solutions, in cases where there exist multiple solutions of structures that produce the same best property, is reflected by the number of distinct answers generated, normalized by the most answers found thus far.

The three baseline methods to compare with are: 1) an exhaustive search (eSearch) containing 1 million random searches, 2) a generalized pattern search^22^, which can be considered as a smarter-than-random guided search (gSearch), 3) a traditional optimization algorithm, specifically the linear programming (LP) is used for linear problems and genetic algorithms (GA) for nonlinear problems. In the exhaustive search, to ensure the constraint satisfaction the Random Interval (details in Method section under random data generation) method is used to generate 1 million realizations of i.i.d. ODF values. The guided search, however, should require far less iterations and we randomize 100 initial starting positions of ODF and for each iteration, the search goes until a local optima is found.

Table 1 shows optimal values of each design function obtained by the aforementioned methods. As we can deduce from this comparison, for linear properties as *Y* and *m*_*s*_, LP always provides a valid solution. It is the nonlinear problems as *E*, *F*_1_ and *F*_2_ that pose a challenge for traditional optimization methods. Exhaustive search gives largely unstable results; one almost has to rely on pure luck to bet on a fair result. Guided search has a tendency to get stuck at local optima, which can sometimes be even worse than the naive exhaustive search (although the running time is much less). Surprisingly, GA has worked poorly on nonlinear problems (we also tried GA on linear problems and the answer is never as good as LP). In fact, for nonlinear problems the guided search is often a better choice than GA. ML methods have been proven successful achieving the best answers throughout all problems, linear and nonlinear.[Fig f5][Fig f6]

We use visualization bars**, s**hown in Fig. 7, to illustrate the performance of different methods in a standardized manner in terms of all three criteria. Figure 7(left) describes the normalized level of optimality achieved by each method (shown by vertical color sticks) for each problem (each grey box). The highest level (rightmost) is defined by the best solution found among five methods, and the lowest level (leftmost) represents the worst answer among the five. The position in between is at linear scale. Therefore how rightward the positions of color sticks represent how good the method is regarding finding the optimum answer.

**Figure 5 f5:**
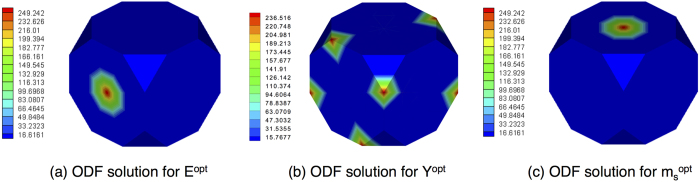
Visualization of ODF solutions to three problems. (**a**) ODF that satisfies the objective function *E*. (**b**) ODF that satisfies the pbjective function *Y*. (**c**) ODF that satisfies the objective function *m*_*s*_.

The minimum of Young’s modulus (*E*^*opt*^) obtained is 85.9878 GPa. Although the property is nonlinear with respect to the ODF values, multiple ODF solutions were not found. The optimal ODF was found to be a single-crystal with (011)[100] orientation, shown in Fig. 5(a). As is evident from the plots, the extremal ODF corresponds to a unique orientation (and symmetric equivalents, if any). Although the ODF ideally is a Dirac delta function at this orientation, a finite ODF value is plotted due to the use of a finite element discretization. In addition, the use of finite elements implies that this is not the exact minimum unless there is a node exactly at the location of the optimal orientation. However, as^10^ pointed out, one can achieve the property bounds accurately with finer discretizations of the Rodrigues space.

Since yield stress (*Y*^*opt*^) is a linear property, the extremal values correspond to single crystals. In our analysis, the maximum value was found to be 353.11 MPa which is equal for two single crystals, close to the 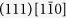 orientation as shown in Fig. 5(b). Similar analysis to find the ODF with maximal magnetostrictive strain (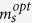) revealed a single-crystal at the 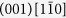 orientation with field induced strain of 1.5498e−04. Note that crystal z–axis is the direction of easy magnetization and of the largest saturation magnetostriction in single crystal galfenol and the optimal crystal, as expected, has this direction aligned with the direction of strain measurement. Figure 5(c) shows the corresponding ODF.

**Figure 6 f6:**
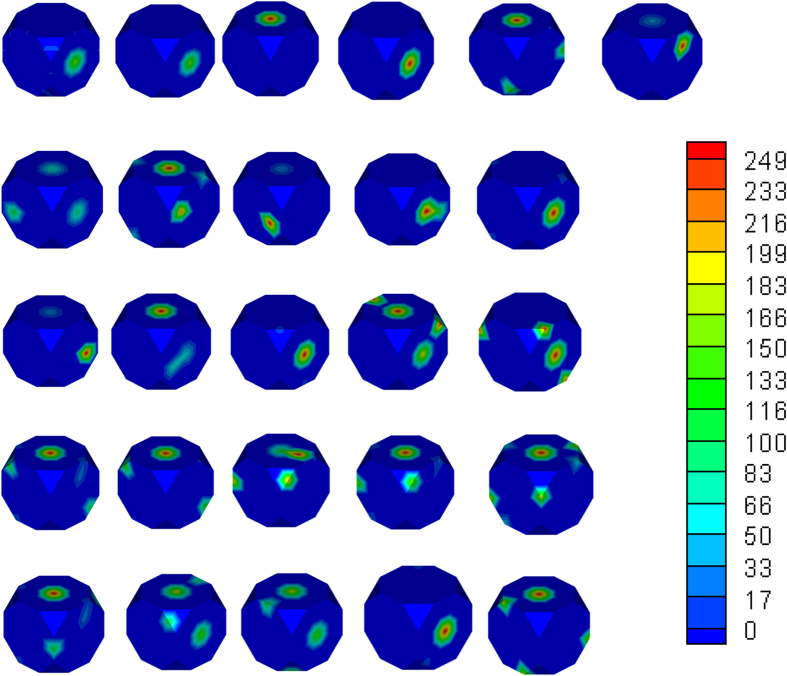
Visualization of ODF solutions to *F*_2_. All 26 cases that maximize the objective function *F*_2_ are shown.

The optimum microstructure for objective function *F*_1_ was also a single-crystal. For problem *F*_2_, however, we get 26 different ODFs which give the same maximum value of 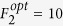. ODF plots for all these solutions are shown in Fig. 6. Most distinctively, these 26 solutions are all polycrystal, taking values on every one of the crystalline orientations. In this sense, the ML method manages to discover complex and irregular compositions of microstructure in the search for extreme values.

**Figure 7:**
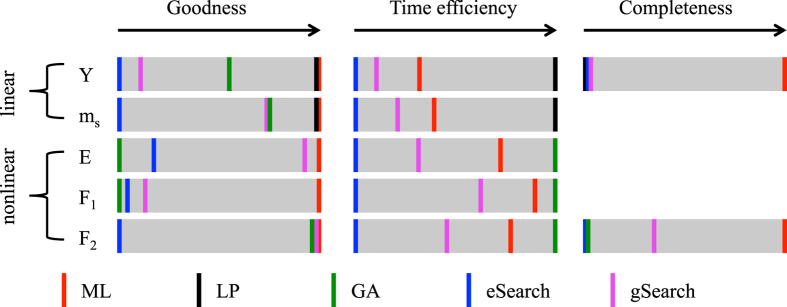
Visualization of method effectiveness. Three criteria are used: goodness of properties (left), time efficiency (middle), and completeness of solution (right).

A running time comparison is made in Table 2. For these problems LP can finish within 1 second and each round of GA normally takes 2 to 3 seconds. LP and GA (with 100 restarts and takes about 200 seconds) are shown in Fig. 7(middle) although not included in Table 2. eSearch conducts 1 million random searches along with 1 million times of function evaluation, and gSearch takes 100 iterations each taking a different random initial point. Fig. 7(middle) illustrates the normalized level of time efficiency produced by each method for each problem. The highest level (rightmost) is defined by the one using the least time in producing its final answer, and the lowest level (leftmost) represents the longest time taken among the five. Since the time varies among methods from a couple of seconds tens of thousands of seconds, the position in between is in a logarithmic scale. eSearch, as imagined, takes the longest time to finish since it explores randomly the space without a proper guidance. ML takes less than 5% of time consumed by eSearch. Compared with gSearch, ML reduced respectively 84.94%, 88.05%, 83.67%, 72.62%, and 78.82% of the running time. The average reduction is 81.62%. The 4-th step of ML framework is the exact same gSearch with reduced variables; this implies that the reduced time comes from the reduced searching space.

Last but not least, the completeness of solution is an important gauge of optimization design. Two of our design problems, the optimization of *Y* and *F*_2_, appear to have multiple solutions that lead to identical optimum answers. The completeness of solutions for each method regarding these two problems is shown in Fig. 7(right). The linear problem has three solutions found by ML that lead to the same *Y*^*opt*^, but only one is found by LP. eSearch and gSearch each also found one solution but according to Table 1 neither of them generates property as good as *Y*^*opt*^. For the nonlinear problem *F*_2_, eSearch and GA each obtained one solution and neither is as good as 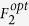. gSearch was able to produce 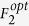, but it only found 9 solutions. Only ML is able to find 26 solutions, the most complete as far as we know.

## Discussion

The selection of materials and geometry to minimize (or maximize) some given property has been a common problem in material science. The optimum answer can be obtained by an exhaustive search among numerous choices in the searching domain bounded by some boundary conditions and constraints. More intelligently and less laboriously, the search can be guided by heuristics, and how the heuristic is designed is discussed by various researchers in the area of searching algorithms and artificial intelligence^23^,^24^. However, searches that are exhaustive or follow simple heuristics suffer from the high dimensionality used in the structure representation, and they often return incomplete solution sets.

Machine learning based dimension reduction techniques are known to be powerful in analyzing variable relationships in large datasets containing high variable dimensions. It is often used for data representation for the sake of reducing storage space as well as computational efforts. Regarding optimization problems, ML methods can be considered to reduce the search space by limiting the number of design variables in search and finding patterns to form a superior searching strategy. The analysis that ML is able to make to a reasonable collection of variable-objective data instances could provide valuable insights towards the variable relations, and answer questions as what variables to search first, what areas are the most promising, what direction would possibly lead to quick convergence, etc.

In this work, we applied the technique to optimize the magnetoelastic properties of Iron–Gallium alloy (Galfenol). The orientation space of BCC Galfenol was discretized using finite elements to obtain a compact representation of design variables. We searched for microstructures with optimal properties, such as low elastic modulus and high magnetostrictive strains, while also exploring possible non–unique solutions for these design cases. We can deduce from the experimental results that linear problems with unique solutions are better solved with LP, which is both fast and accurate. However, multiple solutions are not explored when using LP techniques. GA performed incompetently across all problems despite its advantage in running time efficiency, in that it was never able to produce the best known answers in our problems. ML method was able to achieve a fine balance between accuracy and efficiency, and more importantly, it is able to find the complete set of solutions that none of the other methods can.

## Methods

### BCC crystal plasticity model

A rate-independent single-crystal plasticity model developed in Kothari and Anand^25^ is used to compute the effect of macroscopic strain on the polycrystal. For a material with *α* = 1,…,*N* slip systems defined by ortho-normal vector pairs (***m***^*α*^, ***n***^*α*^) denoting the slip direction and slip plane normal respectively, the constitutive equations relate the following basic fields: the deformation gradient ***F*** which can be decomposed into elastic and plastic parts as ***F*** = ***F***^*e*^***F***^*p*^, the Cauchy stress ***T*** and the slip resistances *s*^*α*^ > 0. In the constitutive equations, the Green elastic strain measure 
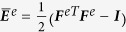
 defined on the relaxed configuration (plastically deformed, unstressed configuration) is utilized. The conjugate stress measure is then defined as 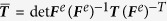 and the stress–strain relation is given by 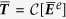 where 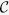 is the fourth-order elasticity tensor. It is assumed that deformation takes place through dislocation glide and the evolution of the plastic flow is given by


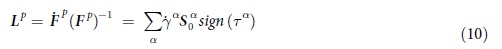


where 
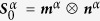
 is the Schmid tensor and 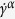 is the plastic shearing rate on the *α*^*th*^ slip system. The resolved stress on the *α*^*th*^ slip system is given by 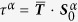. The resolved shear stress 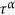 attains a critical value *s*^*α*^ on the systems where slip occurs (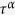 = *s*^*α*^, if 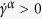). Further, the resolved shear stress does not exceed *s*^*α*^ on the inactive systems with 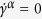. The hardening law for the slip resistance *s*^*α*^ is taken as,


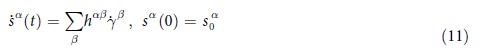


The slip system hardening model is given as:


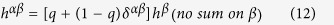


where *h*^*β*^ is a single slip hardening rate, *q* is the latent-hardening ratio and *δ*^*αβ*^ is the Kronecker delta function. The parameter *q* is taken to be 1.0 for coplanar slip systems and 1.4 for non-coplanar slip systems. For the single-slip hardening rate, the following specific form is adopted:


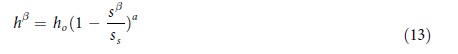


where *h*_o_, *a*, and *s*_*s*_ are slip hardening parameters taken to be identical for all slip systems, with values *h*_o_ = 500 MPa, s_s_ = 350 MPa and *a* = 2.25and for BCC Galfenol single crystals. The initial value of slip system resistance is calibrated as s_*o*_ = 180 *MPa*. Plastic deformation due to crystallographic slip is assumed to occur in the 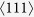 direction, and the possible slip planes are of the {110}, {112}, and {123} type.

### Random data generation

Data that are expected to be seen during the search are simulated and collected before the actual search. For the purpose of supervised learning the training set is in a form of {***X***^(*j*)^,*y*^(*j*)^}, where the ***X***^(*j*)^ and *y*^(*j*)^ are the *j*-th training example of the feature vector and the corresponding class label. In our design problem, ***X*** = [*x*_1_,*x*_2_,…,*x*_*D*_] ⊆ *R*^*D*^ is an instance of structure representation (e.g. the multidimensional ODF) and *y*^(*j*)^ ∈ {−1,1} is an indicator of how good a property generated by the structure is. Given a property function *f*(***X***), supposedly to be maximized, the class label *y*^(*j*)^ = 1 is assigned to input vector ***X***^(*j*)^ if the function value *f*(***X***^(*j*)^) is ‘sufficiently high’. For some algorithms to work well, data from the opposing class might be needed, which indicates sufficiently low property and are assigned a class label of −1. Data quality of the training set is ensured by enforcing randomness and polarization, with the four randomization methods, namely, Random Intervals, Random *k* Intervals, Random Every *k*, and Best-First Assignment, developed to address randomness from different angles and generate samples under the constraints in our problems: 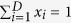 and ***X*** ≥ 0. While one of them (RI) gives the chance of taking values to every variable equally, the others tend to think that a limited number (*k*) of variables are of greater importance than the others, only we don’t know which *k*. After the randomized data generation, a data polarization procedure takes portions of samples from two extreme ends, on top of which the feature learning is devised.

(a) Random Intervals (RI). We consider the unit length 1 is divided into *D* random intervals, or making *D* − 1 random cuts between the interval [0,1], where *D* is the dimension of ***X***, or ODF. Then the length of intervals are randomly assigned to each feature *x*_*i*_.

(b) Random *k* Intervals (RkI). This is similar to *RI* but each time, only *k* intervals are generated and assigned to *k* random dimensions. *k* is iterated from 1 to *D* − 1 with an increasing number of samples generated with regards to *k* (roughly a linear relationship) and then down sampled to 1000 for each iteration, except when *k* = 1, *D* samples exist and are all used.

(c) Random Every *k* (REk). Randomly generate *k* values at a time, continue only when the sum *s* of the current *k* values does not exceed the threshold 1. Update the threshold to the remainder 1 − *s*, and repeat the process until the remainder is sufficiently small. Assign the generated values to a random set of features. *k* is fixed to be 5 in the experiment.

(d) Best-First Assignment (BFA). Randomly pick a feature and assign to it a random value *u*, 0 ≤ *u* ≤ 1. Distribute the remainder 1 − *u* evenly to all other variables so that the constraint is met. Compute the objective function and obtain the function value. Repeat *n* times and continue with whichever gives the best function value. Fix that selected variable and repeat the process to select another variable, and go on until no variable is left.

In terms of operation time, REk and BFA are the slowest since they either operate under a probability of getting valid feature values or involve multiple evaluations of the objective function. In generating random samples for *F*_2_ with a MATLAB implementation, RI takes an average of 0.99 ms per sample, RkI 0.42 ms, REk 12.5 ms, and BFA 12.7 ms. We let RI generate 50,000 data instances and terminate REk and BFA when their generated samples reach 10,000. Thus in total we obtained around 145,000 samples. We then sort according to the objective value and keep the top and bottom 25% of data instances, with one of the extremes desired and the other undesired, each with 36,250 samples.

### Search path refinement via feature selection

The motivation of introducing feature ranking into optimization is to obtain a specialized search path in the form of a sorted order of variables prior to the start of search, so as to improve the searching efficiency. Four supervised feature ranking methods, *χ*^2^, Information Gain, F-score^26^ and SVM-weight (SVM^27^) are employed. They work either through calculating a coefficient (also called filter methods) to characterize the relevance of each feature with the class target, or through building classifiers (also called wrapper methods) with each variable and evaluating the performance. Filter methods are generally faster. The final feature ranking is decided by a voting (majority wins) of the four result sets, and it determines the order of search, that is, the order of variables whose values get updated. In the search to follow, at each iteration only one variable gets updated (by, say, gradient descent), and its value fixated when the objective function stops improving. In this way, prior to the search a search path is determined, which yields greater efficiency. To retain a degree of openness, we conduct multiple searches by shuffling the top 10% of the ranked list, which proves to be critical in obtaining multiple answers for a problem in later experiments.

### Search space reduction via classification schemes

For the purpose of reducing the search region of each variable, in this subroutine we build a rule-based classification tree to learn the most promising region of values for each variable. As in the path refinement activity, the data category with desired function values is represented by the class ‘1’ and the contradictory class is labeled as ‘−1’. This creates a two-class classification problem. We use rule-based classifiers, such as decision trees, because they are easily traversed and thresholds are clearly attained. After a tree is constructed, we look for the leaf nodes with “−1” since our purpose is to minimize *E*. We traverse from the root to each of the “−1” leaf nodes and write down the rules generated along the path. The number of samples covered by the rule and the accuracy of the rule should also be considered. For the *E*-minimize example the most supported rule is: IF *x*_16_ ≤ 0.17565 AND *x*_26_ ≤ 0.20504 AND *x*_14_ ≤ 0.13302 AND *x*_53_ ≥ 0.064713 AND *x*_37_ ≤ 0.004566 THEN “*y* = −1”. Therefore, the searching regions for these variables are modified to *x*_16_ ∈ [0,0.17565], *x*_26_ ∈ [0,0.20504], *x*_14_ ∈ [0,0.13302], *x*_53_ ∈ [0.064713,1], and *x*_37_ ∈ [0,0.004566]. The searching effort is thus reduced to a more concentrated area on these variables. Compared to the original region of [0,1], relatively 83%, 80%, 87%, 6% and 99% of the search region has been reduced.

### Enhanced optimization

A gradient-based line search^28^ is conducted on a ordered list of variables, finding, one variable at a time, the value of it that optimizes the function from a reduced value space. Top-rank shuffling and multi-starting strategies are incorporated so that on each run, the algorithm starts from a randomly generated initial solution in the search space, with a slightly shuffled pre-planned searching order of variables. Optimization becomes a much promising endeavor when the search space is reduced and a pre-planned searching path is deployed.

## Additional Information

**How to cite this article**: Liu, R. *et al.* A predictive machine learning approach for microstructure optimization and materials design. *Sci. Rep.* **5**, 11551; doi: 10.1038/srep11551 (2015).

## Figures and Tables

**Table 1:** Summary of optimal values for all design problems and methods.

**Property**	**Objective**	**eSearch**	**gSearch**	**LP/GA**	**ML**
*E* (GPa)	Min	127.56	89.5667	136.25	**85.9878**
*Y* (MPa)	Max	301	306.54	**353.11**	**353.11**
*m*_*s*_	Max	9.0034e−05	1.3797e−04	**1.5498e−04**	**5498e−04**
*F*_1_	Max	1.0911e−07	1.2609e−07	1.0122e−07	**2.9347e−07**
*F*_2_	Max	9.81	**10**	9.9987	**10**

**Table 2:** Summary of running times (s) for all design problems and methods.

**Property**	**eSearch**	**gSearch**	**ML**	**Percentage of time reduced**
*E*	20054	4718.5	710.4	84.94%
*Y*	20145	7150.5	854.6	88.05%
*m*_*s*_	21302	2588.0	422.5	83.67%
*F*_1_	23225	1179.1	322.8	72.62%
*F*_2_	25431	2783.3	589.4	78.82%
